# Analgesic effect and safety of single-dose intra-articular magnesium after arthroscopic surgery: a systematic review and meta-analysis

**DOI:** 10.1038/srep38024

**Published:** 2016-11-30

**Authors:** Chao Zeng, Yu-sheng Li, Jie Wei, Dong-xing Xie, Xi Xie, Liang-jun Li, Shu-guang Gao, Wei Luo, Yi-lin Xiong, Wen-feng Xiao, Guang-hua Lei

**Affiliations:** 1 Department of Orthopaedics, Xiangya Hospital, Central South University, Changsha, Hunan Province, 410008, China; 2 Health Management Center, Xiangya Hospital, Central South University, Changsha, Hunan Province, 410008, China; 3 Department of Epidemiology and Health Statistics, School of Public Health, Central South University, Changsha, Hunan Province, 410008, China

## Abstract

To examine the analgesic effect and safety of single-dose intra-articular (IA) magnesium (Mg) after arthroscopic surgery. Pubmed, Embase and Cochrane library were searched through in January 2016. Eight RCTs and eight experimental studies were included. The IA Mg exhibited a significantly lower pain score when compared with placebo (MD, −0.41, 95% CI, −0.78 to −0.05, p = 0.03). There was no significant difference between Mg and bupivacaine in terms of pain relief and the time to first analgesic request. Furthermore, statistically significant differences both in pain score (MD, −0.62, 95% CI, −0.81 to −0.42, p < 0.00001) and time to first analgesic request (MD, 6.25, 95% CI, 5.22 to 7.29, p < 0.00001) were observed between Mg plus bupivacaine and bupivacaine alone. There was no statistically significant difference among the various groups with respect to adverse reactions. Most of the included *in vitro* studies reported the chondrocyte protective effect of Mg supplementation. There were also two *in vivo* studies showing the cartilage protective effect of IA Mg. The single-dose IA Mg following arthroscopic surgery was effective in pain relief without increasing adverse reactions, and it could also enhance the analgesic effect of bupivacaine. In addition, Mg seemed to possess the cartilage or chondrocyte protective effect based on experimental studies.

Post-operative pain after arthroscopic surgery, which is commonly performed as a day case, is one of the obstacles delaying hospital discharge and hindering early rehabilitation^1^,^2^. The single-dose intra-articular (IA) analgesic agents has been widely used for pain relief after arthroscopic surgery as a simple and economical approach. Previous meta-analyses have demonstrated that the single-dose IA morphine^3^,^4^, bupivacaine^5^,^6^,^7^, ropivacaine^8^, clonidine^9^ or the combination of morphine and bupivacaine^10^,^11^ were effective in pain relief. However, it is commonly known that IA local anesthetics must be used cautiously due to the concern of chondrotoxicity^12^, especially for bupivacaine^13^,^14^,^15^,^16^,^17^,^18^,^19^, levobupivacaine^13^,^14^,^19^, ropivacaine^13^,^14^,^16^,^17^, mepivacaine^17^ and lidocaine^13^,^16^,^18^,^20^.

Magnesium (Mg), a physiologic N-methyl-D-aspartate (NMDA) receptor antagonist, could be effective in postoperative analgesia^21^,^22^. More importantly, Mg has been proved to be able to enhance the chondrocyte viability either alone or in combination with a local anesthetic (rather than a local anesthetic alone), and it was therefore proposed to be a potential alternative IA analgesic agent due to its effect on chondrotoxicity reduction^13^,^14^. However, it remains to be a controversial issue whether the single-dose IA Mg after arthroscopic surgery is effective in pain relief when compared with placebo, or whether the IA Mg exhibits a comparable effect when compared with bupivacaine, which is the most widely used local analgesic at present^23^,^24^,^25^,^26^,^27^,^28^,^29^,^30^. In addition, there is also a great significance for clinical practice to explore whether IA Mg can amplify the analgesic effect of bupivacaine and reduce the chondrotoxicity of local anesthetics. Furthermore, some broader issues related to the analgesic ability and chondrocyte protective effect of IA Mg in other settings, such as osteoarthritis, are to be addressed.

Therefore, the objectives of this systematic review and meta-analysis were to investigate the analgesic effect of single-dose IA based on the following comparisons: (1) Mg versus placebo, (2) Mg versus bupivacaine and (3) Mg plus bupivacaine versus bupivacaine alone, after arthroscopic surgery. Another objective was to assess the protective effect of Mg on chondrocyte or cartilage based on randomized controlled trials (RCTs), *in vitro* and *in vivo* studies.

## Methods

### Literature search

This meta-analysis was in accord with the Preferred Reporting Items for Systematic review and Meta-analyses statement^31^. The electronic databases of Pubmed, Embase and Cochrane library were searched through in January 2016 using a series of logic combination of keywords and text words related to arthroscopic knee surgery, magnesium and randomized controlled trials (RCTs) (Appendix 1). The Pubmed and Embase databases were also searched through in January 2016 to retrieve *in vitro* and *in vivo* experimental studies of Mg supplementation (Appendix 1). No restriction was imposed, and the references of the retrieved articles and reviews were evaluated.

### Study selection

Two researchers reviewed all the retrieved titles, abstracts and full texts independently. Disagreements were resolved through discussions and/or consultations to a thrid researcher. All of the eligible trials must meet the following criteria: (1) patients undergone arthroscopic surgery; (2) including a treatment group of single-dose IA Mg or Mg plus bupivacaine for postoperative pain relief; (3) including a controlled group of IA placebo or bupivacaine alone, (4) other interventions should be balanced between comparative groups, (5) RCTs. The exclusion criteria for this study were: (1) case series, reviews, protocols or vitro studies; (2) non-RCTs; (3) abstract or full text was not available; (4) no IA injection; (5) meeting abstract.

### Data extraction

The available information and outcomes of each included study were extracted by two independent researchers. The retained data included the first author, year of publication, size of each group, doses of intervention, follow-up time points, type of operation, type of anesthesia and injection time. The Cochrane risk of bias table was used to assess the methodological quality of the included RCTs. A total of seven potential risks of bias were evaluated: random sequence generation, allocation concealment, blinding of participants, blinding of outcome assessment, incomplete outcome data, selective reporting and other bias (mainly including the conflict of interests)^32^. Each risk item was evaluated based on a three-level rating system: low risk, unclear risk and high risk. Studies with three or more unclear or high risk of bias were considered as poor methodological quality.

The primary outcome of interests for this study was the effects of IA Mg and Mg plus bupivacaine on postoperative pain control. If a study reported multiple pain scales, the highest one on the hierarchy of pain scale related to the outcomes was adopted, as described by Jüni and colleagues^33^. The secondary outcomes of interest were the time interval to the first request of analgesia and the side effects. If data was presented in figures, the GetData software would be used (http://getdata-graph-digitizer.com/index.php) to extract data from the figures. If the outcomes were reported in terms of the median and the range or interquartile range, the median would be considered as mean and the standard deviation would be estimated by the range or interquartile range^32^.

### Statistical analyses

The outcome measures investigated in this meta-analysis were postoperative pain intensity and the time to first request of analgesia. Due to the low incidence of adverse reactions, only qualitative analysis were conducted on relevant adverse effects. Firstly, we calculated the overall mean difference (MD) and its corresponding 95% confidence interval (CI) between comparative groups for postoperative pain intensity at last follow up. Then a subgroup analysis were conducted by calculating the MD at different time points of follow up. If all included trials reported the pain intensity at a same postoperative time point, an overall MD between comparative groups for pain intensity was calculated at this time point of follow up. We also calculated the overall MD and its related 95% CI for the time to first request of analgesia. The heterogeneity of effect size across trials was tested by Q statistics (p < 0.05 was considered heterogeneous). If there was a significant heterogeneity among the studies, the random-effects model was used; otherwise, the fixed effects model was acceptable. We also examined the I^2^ statistic, which measures the percentage of the total variation across studies due to heterogeneity (I^2^ > 50 was considered heterogeneous). We further conducted sensitivity analysis to explore possible explanations for heterogeneity and to examine the influence of various exclusion criteria on the overall MD.

Begg’s tests^34^ and funnel plots were performed to assess the publication bias. Statistical analyses were performed using Review Manager 5 software (RevMan 5, The Cochrane Collaboration, Oxford, UK) and STATA version 11.0 (StataCorp LP, College Station, Texas). A p value less than 0.05 was considered to be statistically significant.

## Results

### Search results and selected articles

**Figure 1:**
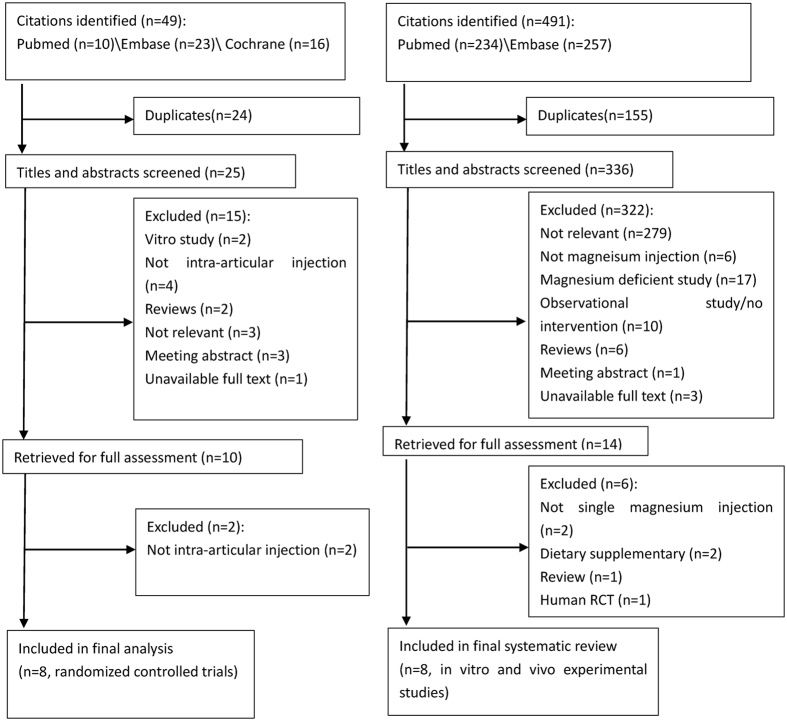
Flow diagram of included studies.

The literature search retrieved a total of 49 citations for potential RCTs examining the analgesic effect of single-dose IA Mg after arthroscopic surgery and 491 citations for potential *in vitro* and *in vivo* experimental studies of Mg supplementation, respectively. Two evaluators screened the titles/abstracts and full-texts of all eligible articles. Eventually, 8 RCTs^23^,^24^,^25^,^26^,^27^,^28^ (513 patients) and 8 experimental studies^13^,^14^,^29^,^30^,^35^,^36^,^37^,^38^,^39^,^40^ (6 *in vitro* and 2 *in vivo* studies) were qualified for final analysis (Fig. 1). The characteristics of the included RCTs are presented in Table 1. The characteristics and results of the included *in vitro* and *in vivo* experimental studies are listed in Table 2. According to the Cochrane risk of bias table (Appendix 2), three trials^25^,^27^,^29^ did not describe their random sequence generation (high risk of selection bias); six trials^24^,^25^,^26^,^28^,^29^,^30^ did not design a clear allocation concealment plan (unclear or high risk of selection bias); all trials adopted the double-blind method except one^27^ which did not describe the blinding method of participants (high risk of performance bias); one study^30^ was rated as unclear risk for both incomplete data and selective reporting; six studies^23^,^24^,^25^,^26^,^27^,^28^ did not present a competing interest statement (unclear risk of other bias). In addition, three trials^25^,^27^,^30^ were rated as low methodological quality for having three or more unclear or high risk of bias.

### Effects of pain relief

#### Mg versus placebo

**Figure 2:**
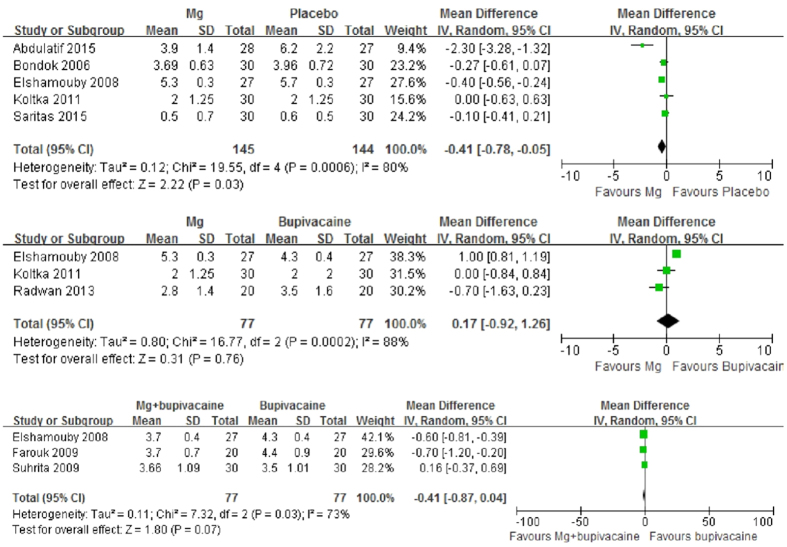
Forest plot of pain intensity at the last follow-up time point.

Five trials^23^,^24^,^27^,^29^,^30^ were eligible for assessing the postoperative pain score of IA Mg versus placebo after arthroscopic surgery at the last follow-up time point (24 or 48 hours). The combined results showed a significantly lower pain score of Mg (MD, −0.41, 95% CI, −0.78 to −0.05, p = 0.03). Substantial heterogeneity was observed (p = 0.0006, I^2^ = 80%) (Fig. 2). Sensitivity analyses after excluding studies with poor methodological quality and studies involving femoral nerve block in each group or involving other types of surgery rather than single knee meniscectomy all showed positive results with reduced heterogeneity (p > 0.05) (Appendix 3). One of the studies reported data with median and range was further excluded in sensitivity analysis, and the result was similar. Meanwhile, the positive results of subgroup analyses remained unchanged at different follow-up time points (2, 12 or 24 hours) (Appendix 4). Begg’s rank correlation test suggested no evidence of publication bias among included studies (p = 0.806).

#### Mg versus bupivacaine

Three trials were^24^,^27^,^28^ eligible for assessing the postoperative pain score of IA Mg versus bupivacaine (including levobupivacaine) after arthroscopic surgery at the last follow-up time point (24 or 48 hours). The combined results showed no significant difference between Mg and bupivacaine (MD, 0.17, 95% CI, −0.92 to 1.26, p = 0.76). Substantial heterogeneity was observed (p = 0.0002, I^2^ = 88%) (Fig. 2). Sensitivity analyses after excluding studies with poor methodological quality, or studies involving levobupivacaine rather than bupivacaine, or data reported by median and range showed the same results (Appendix 3). Meanwhile, the results of subgroup analyses remained to be insignificant at different follow-up time points (1, 2, 4, 12 or 24 hours) (Appendix 4). Begg’s rank correlation test suggested no evidence of publication bias among included studies (p = 0.296).

#### Mg plus bupivacaine versus bupivacaine alone

Three trials^24^,^25^,^26^ were eligible for assessing the postoperative pain score of IA Mg plus bupivacaine versus bupivacaine alone after arthroscopic surgery at the last follow-up time point (18 or 24 hours). The combined results showed that the effect of pain relief of Mg plus bupivacaine approached to the significant level when compared with bupivacaine alone (MD, −0.41, 95% CI, −0.87 to 0.04, p = 0.07). Substantial heterogeneity was observed (p = 0.03, I^2^ = 73%) (Fig. 2). Sensitivity analyses after excluding studies involving other types of surgery rather than single knee meniscectomy reached a significant result (MD, −0.62, 95% CI, −0.81 to −0.42, p < 0.00001) while heterogeneity disappeared (p = 0.72, I^2^ = 0%) (Appendix 3). The results of subgroup analyses remained to be significant at different follow-up time points (1 or 2 hours) (Appendix 4). Begg’s rank correlation test suggested no evidence of publication bias among included studies (p = 0.296).

### Time to first analgesic request

#### Mg versus placebo

**Figure 3:**
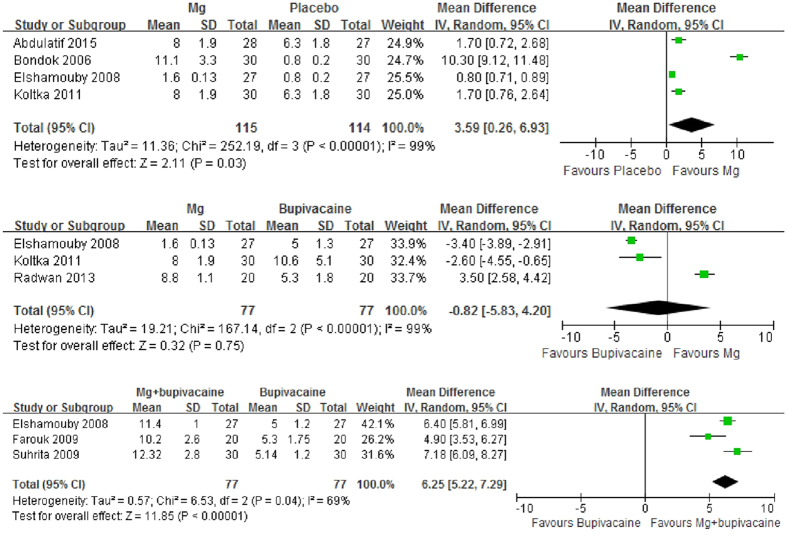
Forest plot of time to first analgesic request.

Four trials^23^,^24^,^27^,^30^ were eligible for assessing the time interval before the first request for analgesic medication of IA Mg versus placebo after arthroscopic surgery. The combined results showed a significantly longer time interval of Mg (MD, 3.59, 95% CI, 0.26 to 6.93, p = 0.03). Substantial heterogeneity was observed (p < 0.00001, I^2^ = 99%) (Fig. 3). However, sensitivity analyses after excluding studies with poor methodological quality and studies involving femoral nerve block in each group or involving other types of surgery rather than single knee meniscectomy all denied such positive results (p > 0.05) (Appendix 3). Begg’s rank correlation test suggested no evidence of publication bias among included studies (p = 0.308).

#### Mg versus bupivacaine

Three trials^24^,^27^,^28^ were eligible for assessing the time interval before the first request for analgesic medication of IA Mg versus bupivaciane after arthroscopic surgery. The combined results showed no significant difference between Mg and bupivaciane (MD, −0.82, 95% CI, −5.83 to 4.20, p = 0.75). Substantial heterogeneity was observed (p < 0.00001, I^2^ = 99%) (Fig. 3). Sensitivity analyses after excluding studies with poor methodological quality or studies involving levobupivacaine rather than bupivacaine reached the same results (p > 0.05) (Appendix 3). Begg’s rank correlation test suggested no evidence of publication bias among included studies (p = 1.000).

#### Mg plus bupivacaine versus bupivacaine alone

Four trials^24^,^25^,^26^ were eligible for assessing the time interval before the first request for analgesic medication of IA Mg plus bupivacaine versus bupivacaine alone after arthroscopic surgery. The combined results showed a significantly longer time interval of Mg plus bupivacaine (MD, 6.25, 95% CI, 5.22 to 7.29, p < 0.00001). Substantial heterogeneity was observed (p = 0.04, I^2^ = 69%) (Fig. 3). Sensitivity analyses after excluding studies with poor methodological quality reached the same positive results with reduced heterogeneity (p = 0.22, I^2^ = 34%) (Appendix 3). Begg’s rank correlation test suggested no evidence of publication bias among included studies (p = 1.000).

### Safety

#### Adverse reactions reported in RCTs

Only four included RCTs^27^,^28^,^29^,^30^ reported adverse reactions (including knee effusion, nausea, vomiting, flushing, shivering, hypotension, bradycardia and drowsiness), as illustrated in Table 2. There was no statistically significant difference between comparable groups (including IA Mg versus placebo) in each trial.

#### Results of *in vitro* and *in vivo* experimental studies of Mg supplementation

The characteristics and analysis results of the included experimental studies were reported in Table 3. In comparison with local analgesics, Mg-free or low-level Mg medium, Mg exhibited a clear chondrocyte protective effect, which is exerted by increasing the number of attached chondrocytes^35^, enhancing chondrocyte proliferation and redifferentiation^36^, and reducing chondrocyte toxicity (increasing viability)^13^,^14^. However, there are two studies^37^,^38^ suggesting that high level of Mg (50 and 100 mM) or extra-high concentration of Mg could decrease the extracellular matrix protein, such as collagen and glycosaminoglycan. Only two *in vivo* studies^39^,^40^ further revealed that IA Mg attenuated the development of osteoarthritis in the rat model of collagenase-induced osteoarthritis and promoted cartilage formation of synovial mesenchymal stem cells in the rabbit osteochondral defect model. It should be highlighted that the limited number of *in vivo* studies retrieved in this area may affect the reliability of any conclusion obtained in this respect.

## Discussion

This systematic review and meta-analysis was performed on a total of 8 RCTs (published 2006 to 2015) and 8 *in vitro* and *in vivo* experimental studies. The most important finding of the present study is that the administration of single-dose IA Mg at the end of arthroscopic surgery was effective in pain relief without increasing adverse reactions when compared with placebo, and exhibited a comparable analgesic effect in comparison with bupivacaine. In addition, IA Mg could enhance the analgesic effect of bupivacaine. Another important finding is that Mg seemed to possess cartilage or chondrocyte protective effects according to the included experimental studies. Thus, IA Mg should perhaps be considered as an alternative to local anesthetics for pain relief after arthroscopic surgery. However, the optimal concentration and dosage of IA Mg still needs to be further explored.

Mg is a physiological antagonist of N-methyl-D-aspartate (NMDA) receptor, which is essential to the development and functionality of the nervous system; it serves as a target for the treatment of cognitive impairment, depression, schizophrenia and pain^41^,^42^,^43^,^44^,^45^. Mg could be effective in pain relief by exerting the antinociceptive effect^46^, inhibiting TNF-α^47^, and modulating hypesthesia and hyperalgesia^48^,^49^, by blocking the NMDA receptor. The pain relief effect of Mg has been illustrated by several studies in many cases, such as postoperative sore throat^50^, tourniquet pain^51^, major non-laparoscopic gastrointestinal surgery^52^, diabetic neuropathic pain^53^, cardiac surgery^54^, and cancer-related neuropathic pain^55^. The present study also demonstrated that the single-dose IA Mg was effective in pain relief after arthroscopic surgery.

Several studies, including our previous surveys, have revealed that dietary and serum Mg were negatively associated with the prevalence of knee osteoarthritis^56^,^57^,^58^,^59^, indicating that Mg could possess the cartilage or chondrocyte protective effects. In addition to the included experimental studies (Table 3), Mg was reported to be able to regulate the level of sex determining region Y-box 9, which plays a vital role of cartilage growth plates and is required in the successive steps of chondrogenesis^60^. Furthermore, calcium is critical to the regulation of many cellular physiological functions; the overload of calcium is detrimental to the mitochondrial function^61^. Thus, the inhibition of NMDA receptor by Mg could also decrease the entry of extra-cellular calcium into cells, and thereby exhibits the chondrocyte protective effects. In combination with the findings of the present study, it seems that IA Mg not only can be regarded as an alternative to local analgesics relying on its potential chondrotoxicity, but also shows the cartilage and chondrocyte protective effects. Previous *in vitro* studies have indicated that local anesthetics such as bupivacaine, lidocaine and ropivacaine exert chondrotoxicity by reducing the chondrocyte viability^13^,^14^. The cell death or IA crystal formation caused by mitochondrial DNA damage in chondrocytes could possibly explain the potential toxicity of local anesthetics^62^. However, the exact mechanisms still needs to be further explored.

### Strengths

The present study has several strengths. Firstly, this is the first meta-analysis that examined the analgesic effects of single-dose IA Mg after arthroscopic surgery and demonstrated its effectiveness and safety. Secondly, this study included both *in vitro* and *in vivo* experimental studies to elucidate the potential cartilage and chondrocyte protective effects of Mg supplementation. Thirdly, a comprehensive literature search was performed in several major databases to cover as many eligible RCTs or experimental studies as possible. Thus, the chance of missing any relevant study was fairly low. Lastly, the sensitivity and subgroup analyses supported the robustness of most findings.

### Limitations

Limitations of the present study should also be acknowledged. Firstly, the number of retrieved RCTs was limited and the sample size of each trial was relatively small. This may bias the results. Secondly, a variety of factors may contribute to the heterogeneity of some indexes and affect the results, including the differences in the types of surgery, the dosage of Mg, the setting of follow-up time point, the ages of patients, the severity of pain and the unequal indications for arthroscopy. Especially, the selected dosages of IA Mg were ranged from 500 to 1000 mg, which required to be further investigated to determine the optimal dosage and to demonstrate the safety as the dosage increases. After all, two studies^37^,^38^ suggested that high level of Mg (50 and 100 mM) or extra-high concentration of Mg may not always be beneficial. Thirdly, because of the limited number of included RCTs, this study was unable to compare IA Mg with other local analgesics such as morphine and ropivacaine, which had been demonstrated effective after arthroscopic surgery by our previous meta-analyses^3^,^8^. However, other relevant studies suggested that Mg exhibited the similar analgesic effects when compared with morphine and amplified the effect of morphine or ropivacaine^22^,^63^,^64^,^65^,^66^,^67^. Finally, the limited retrieval of *in vivo* studies (one was about the collagen induced OA model, which is a severe model; the other one was related to stem cells) in the related area may limit the conclusion that Mg supplementation possess the chondroprotective effect.

## Conclusion

Single-dose IA Mg at the end of arthroscopic surgery was effective in pain relief without increasing adverse reactions, and it could also enhance the analgesic effect of bupivacaine. In addition, Mg seemed to exhibit the cartilage or chondrocyte protective effect according to the experimental studies. Perhaps IA Mg should be considered as an alternative to local anesthetics after arthroscopic surgery. However, the optimal concentration and dosage of IA Mg still needs to be explored in further fully powered RCTs, to fully address this point.

## Additional Information

**How to cite this article**: Zeng, C. *et al*. Analgesic effect and safety of single-dose intra-articular magnesium after arthroscopic surgery: a systematic review and meta-analysis. *Sci. Rep.* **6**, 38024; doi: 10.1038/srep38024 (2016).

**Publisher's note:** Springer Nature remains neutral with regard to jurisdictional claims in published maps and institutional affiliations.

## Supplementary Material

Supplementary Information

## Figures and Tables

**Table 1:** Characteristics of the included 8 randomized controlled trials.

Study	Number	Dosage	Time	Type of operation	Type of anesthesia	Injection time
Bondok^23^	Mg: 30	500 mg (10 ml)	1, 2, 6, 8, 12, 18, 24 h	Arthroscopic knee meniscectomy	General	At the end of the surgery
	Placebo: 30	(10 ml)				
Elshamouby^24^	Mg: 27	1000 mg (20 ml)	1, 2, 4, 6, 8, 12, 24 h	Arthroscopic knee meniscectomy	General	At the end of the surgery
	Placebo: 27	(20 ml)				
	B: 27	0.25% (20 ml)				
	Mg plus B: 27	1000 mg plus 0.25% (20 ml)				
Farouk^25^	Mg plus B: 20	150 mg plus 0.25% (20 ml)	1, 2, 24 h	Arthroscopic knee meniscectomy	General	At the end of the surgery
	B: 20	0.25% (20 ml)				
Suhrita^30^	Mg plus B: 30	500 mg plus 0.25% (20 ml)	1, 2, 6, 10, 14, 18 h	Arthroscopic knee meniscectomy and ligament repair	General	At the end of the surgery
	B: 30	0.25% (20 ml)				
Koltka^26^	Mg: 30	500 mg (20 ml)	1, 2, 4, 12, 24, 48 h	Arthroscopic knee meniscectomy	General	At the end of the surgery
	Placebo: 30	(20 ml)				
	LB: 30	0.5% (20 ml)				
Radwan^27^	Mg: 20	800 mg (20 ml)	1, 2, 4, 6, 12, 24 h	Arthroscopic knee meniscectomy	General	At the end of the surgery
	B: 20	0.5% (20 ml)				
Saritas^28^	Mg: 30	100 mg/ml (10 ml)	1, 2, 6, 8, 12, 18, 24 h	Arthroscopic rotator cuff repair	General	At the end of the surgery
	Placebo: 30	(10 ml)				
Abdulatif^29^	Mg: 28	1000 mg (20 ml)	2, 4, 6, 12, 24 h	Arthroscopic ACL reconstruction	General	At the end of the surgery
	Placebo: 27	(20 ml)				

Mg, magnesium sulphate; B, bupivacaine; M, morphine; LB, levobupivacaine; h, hour; ACL, anterior cruciate ligament.

**Table 2:** Adverse reactions reported in the included randomized controlled trials.

Studies	Groups (number)	Adverse reactions (person-time)
Koltka^26^	Mg (30)	Knee effusion (1)
	Placebo (30)	Knee effusion (1)
	LB (30)	Knee effusion (1)
Radwan^27^	Mg (20)	Nausea (3), vomiting (2), flushing (2)
	B (20)	Nausea (2), vomiting (2)
Saritas^28^	Mg (30)	Shivering (12)
	Placebo (30)	Shivering (10)
Abdulatif^29^	Mg (28)	Hypotension (1), bradycardia (1),
	Placebo (27)	Hypotension (1), bradycardia (2), drowsiness (1)

Mg, magnesium; LB, levobupivacaine; B, bupivacaine.

**Table 3:** Characteristics and results of the included 8 *in vitro* and vivo experimental studies of magnesium supplementation.

Study	Mg supplementation	Control group	Chondrocyte or animal model	Results of Mg supplementation
*In vitro* studies				
Egerbacher^35^	MgCl22 (0.0612 mg/ml)	Mg-free medium	Quinolone-treated horse and dog chondrocytes	The number of attached cells increased to 40–70% that of control group (threefold dose led to better results); Cell proliferation did not increase
	MgSO4 (0.0488 mg.ml)			
	MgCl2 (0.0612*3 mg/ml)			
	MgSO4 (0.0488*3 mg/ml)			
Feyerabend^36^	MgSO4 (1, 2, 5, 10, 15, 20, 25, 30 mM)	Without adding MgSO4	Human articular chondrocytes	Enhanced chondrocyte proliferation and redifferentiation (dosage dependent); Increased growth factor effectiveness
Baker^12^	MgSO4 (10%, 20%, 50%)	Placebo	Normal human chondrocytes	MgSO4 alone was no more toxic than placebo; MgSO4 in combination with a local anesthetic reduced chondrocyte toxicity compared with a local anesthetic alone
	Lidocaine (2%) plus MgSO4 (10%, 20%, 50%)	Lidocaine (2%)		
	Levobupivacaine (0.5%) plus MgSO4 (10%, 20%, 50%)	Levobupivacaine (0.5%)		
	Bupivacaine (0.5%) plus MgSO4 (10%, 20%, 50%)	Bupivacaine (0.5%)		
	Ropivacaine (0.75%) plus MgSO4 (10%, 20%, 50%)	Ropivacaine (0.75%)		
Baker^13^	MgSO4 (10%)	Levobupivacaine (0.13%, 0.25%, 0.5%)	Normal human chondrocytes	No significant difference in chondrocyte viability between MgSO4 and placebo; With the exception of 0.13% levobupivacaine, all local anesthetics showed significantly greater toxic effects than MgSO4
		Bupivacaine (0.13%, 0.25%, 0.5%)		
		Ropivacaine (0.19%, 0.38%, 0.75%)		
		placebo		
Hagandora^37^	MgCl2 (20, 50, 100 mM)	Baseline Mg concentration (0.8 mM)	Goat costal chondrocytes (scaffoldless approach)	Collagen and glycosaminoglycan content of the 50 and 100 mM MgCl2 and MgSO4 constructs was significantly lower than the control
	MgSO4 (20, 50, 100 mM) in addition to the baseline Mg concentration (0.8 mM)			
Dou^38^	MgCl2 (10, 20, 30 mM)	Blank control	Knee chondrocytes of Wuzhishan miniature pigs	In 2D culture, low concentrations of Mg ions enhanced excretion of extracellular matrix, whereas extra-high concentration of Mg inhibited the gene expression
*In vivo* studies				
Lee^39^	MgSO4 (500 μg/0.1 ml) twice a week for 5 weeks	Placebo	Rat model of collagenase-induced osteoarthritis	Local intra-articular MgSO4 attenuates the development of osteoarthritis and reduces nociception
Shimaya^40^	Synovial mesenchymal stem cells inμl PBS with 1 or 10 mM Mg	Synovial mesenchymal stem cells inμl PBS	Rabbit osteochondral defect model	Mg promoted cartilage formation of synovial mesenchymal stem cells

Mg, magnesium; MgCl2, magnesium chloride; MgSO4, magnesium sulfate; mM, millimole; PBS, phosphate buffered saline.
