# Supplementary material for: Analgesic effect and safety of single-dose intra-articular magnesium after arthroscopic surgery: a systematic review and meta-analysis
Source: Sci Rep. 2016 Nov 30;6:38024. doi: 10.1038/srep38024 (PMC5128786; doi:10.1038/srep38024)
Supplement: Supplementary Information [file srep38024-s1.pdf]

Analgesic effect and safety of single-dose intra-articular magnesium after arthroscopic surgery: a systematic review and meta-analysis

Chao Zeng[1], Yu-sheng Li[1], Jie Wei[2,3], Dong-xing Xie[1], Xi Xie[1], Liang-jun Li[1], Shu-guang Gao[1], Wei Luo[1], Yi-lin Xiong[1], Wen-feng Xiao[1], Guang-hua Lei[1]*

Appendix 1-Search strategies

Randomized controlled trials:

Pubmed (10)

1. Arthroscopy [tiab] or arthroscopic [tiab] or arthroscope [tiab] or arthrosp* [tiab]
2. Arthroscopy [Mesh]
3. 1 or 2
4. "Magnesium"[Mesh]
5. “Mg”[tiab] or Magnesium[tiab]
6. 4 or 5
7. randomized[tiab]
8. placebo[tiab]
9. controlled[tiab]
10. random*[tiab]
11. trial*[tiab]
12. groups[tiab]
13. ((singl*[tiab] or doubl*[tiab] or tripl*[tiab]) and (mask*[tiab] or blind*[tiab]))
14. Or/7-13
15. 3 and 6 and 14

EMBASE (23)

1. (Arthroscopy or arthroscopic or arthroscope or arthrosp*):ti,ab
2. ‘Arthroscopy’/exp
3. 1 or 2
4. Magnesium:ti,ab
5. ‘Magnesium’/exp
6. 4 or 5
7. (random* or control* or trial* or placebo):ti,ab
8. 3 and 6 and 7

Cochrane library (16)

1. MeSH descriptor Arthroscopy explode all trees
2. (Arthroscopy or arthroscopic or arthroscope or arthrosp*):ti,ab,kw
3. 1 or 2
4. MeSH descriptor Magnesium explode all trees
5. Magnesium:ti,ab,kw

6. 4 or 5
7. 3 and 6

In vitro and in vivo experimental studies:
Pubmed (234)
1. "Chondrocytes"[Mesh]
2. Chondrocyt*[tiab] or Chondroblast*[tiab]
3. "Cartilage"[Mesh] or cartilage[tiab]
4. 1 or 2 or 3
5. "Magnesium"[Mesh]
6. “Mg”[tiab] or Magnesium[tiab]
7. 5 or 6
8. 4 and 7

Embase (257)
1. ‘Chondrocytes’/exp
2. (Chondrocyt* or Chondroblast*):ti,ab
3. ‘Cartilage’/exp
4. Cartilage:ti,ab
5. 1 or 2 or 3 or 4
6. Magnesium:ti,ab
7. ‘Magnesium’/exp
8. 6 or 7
9. 5 and 8

Appendix 2 Methodological quality of the included studies

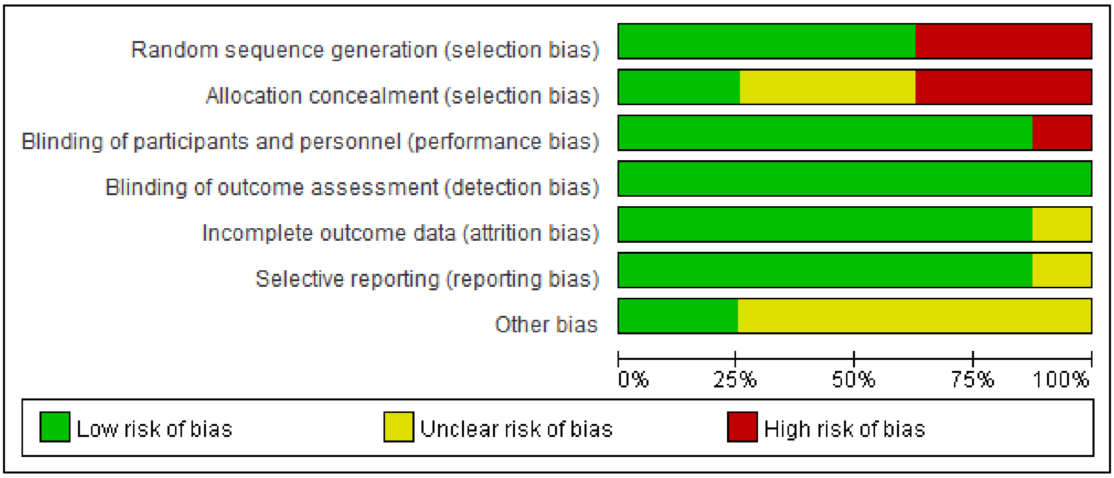

Figure 1 Risk of bias graph: review authors' judgements about each risk of bias item presented as percentages across all included studies.

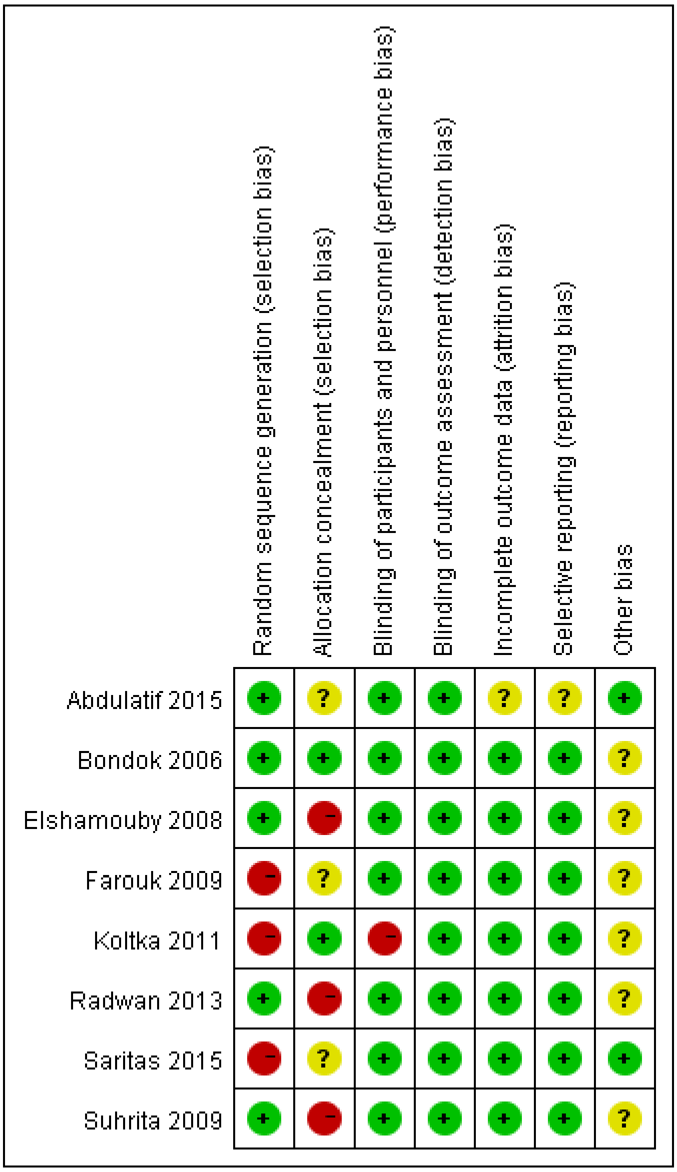

Figure 2 Risk of bias summary: review authors' judgements about each risk of bias item for each included study.

Appendix 3 Sensitivity analyses

Table sensitivity analyses

Comparison	Mg vs placebo		Mg vs bupivacaine		Mg+bupivacaine vs buvacaine	
	MD [95%CI]	Heterogeneity (P/I^2)	MD [95%CI]	Heterogeneity (P/I^2)	MD [95%CI]	Heterogeneity (P/I^2)
Pain intensity at last follow up						
Excluded studies with poor methodological quality	-0.33 [-0.46, -0.20]	0.22/33%	0.21 [-1.45, 1.88]	<0.001/92%	-0.26 [-1.00, 0.48]	0.009/85%
Excluded studies with femoral nerve block in each group	-0.31 [-0.44, -0.18]	0.26/25%	-	-	-	-
Excluded study with levobupivacaine rather than bupivacaine as the control group	-	-	0.21 [-1.45, 1.88]	<0.001/92%	-	-
Excluded study with other type of surgery rather than single knee meniscectomy	-0.26 [-1.00, 0.48]	0.009/85%	-	-	-0.62 [-0.81, -0.42]	0.72/0%
Excluded study which reported data by median and range	-0.50 [-0.92, -0.09]	<0.001/84%	0.21 [-1.45, 1.88]	<0.001/92%	-	-
Time to first request of analgesic						
Excluded studies with poor methodological quality	5.53 [-3.78, 14.84]	<0.001/100%	0.04 [-6.72, 6.80]	<0.001/99%	6.58 [6.06, 7.09]	0.22/34%
Excluded studies with femoral nerve block in each group	4.24 [-0.53, 9.01]	<0.001/99%	-	-	-	-
Excluded study with	-	-	0.04 [-6.72, 6.80]	<0.001/99%	-	-

levobupivacaine rather than bupivacaine as the control group						
Excluded study with other type of surgery rather than single knee meniscectomy	4.24 [-0.53, 9.01]	<0.001/99%	-	-	5.78 [4.34, 7.23]	0.05/74%

Mg, magnesium.

Appendix 4-Subgroup analyses

Study or Subgroup	Mg Mean	Mg SD	Mg Total	Placebo Mean	Placebo SD	Placebo Total	Weight	Mean Difference IV, Random, 95% CI
**1.4.1 Postoperative 2 h**								
Abdulatif 2015	1.8	1	28	2.1	0.8	27	20.0%	-0.30 [-0.78, 0.18]
Bondok 2006	2.2	0.69	30	5.9	1.12	30	20.1%	-3.70 [-4.17, -3.23]
Elshamouby 2008	5.4	0.4	27	5.8	0.5	27	20.5%	-0.40 [-0.64, -0.16]
Koltka 2011	3	1.25	30	5	1.5	30	19.4%	-2.00 [-2.70, -1.30]
Saritas 2015	3.7	0.7	30	4.9	1.2	30	20.0%	-1.20 [-1.70, -0.70]
**Subtotal (95% CI)**			**145**			**144**	**100.0%**	**-1.51 [-2.77, -0.25]**
Heterogeneity: Tau² = 2.00; Chi² = 166.48, df = 4 (P < 0.00001); I² = 98%								
Test for overall effect: Z = 2.35 (P = 0.02)								
**1.4.2 Postoperative 12 h**								
Abdulatif 2015	4.5	2.1	28	5.9	1.7	27	14.4%	-1.40 [-2.41, -0.39]
Bondok 2006	3.51	1.13	30	4.19	0.71	30	21.2%	-0.68 [-1.16, -0.20]
Elshamouby 2008	5.1	0.3	27	5.5	0.4	27	24.0%	-0.40 [-0.59, -0.21]
Koltka 2011	3	1	30	4	1.5	30	19.1%	-1.00 [-1.65, -0.35]
Saritas 2015	1.5	0.6	30	3.3	1.2	30	21.2%	-1.80 [-2.28, -1.32]
**Subtotal (95% CI)**			**145**			**144**	**100.0%**	**-1.02 [-1.61, -0.42]**
Heterogeneity: Tau² = 0.38; Chi² = 32.05, df = 4 (P < 0.00001); I² = 88%								
Test for overall effect: Z = 3.34 (P = 0.0008)								
**1.4.3 Postoperative 24 h**								
Abdulatif 2015	3.9	1.4	28	6.2	2.2	27	14.2%	-2.30 [-3.28, -1.32]
Bondok 2006	3.69	0.63	30	3.96	0.72	30	21.4%	-0.27 [-0.61, 0.07]
Elshamouby 2008	5.3	0.3	27	5.7	0.3	27	22.7%	-0.40 [-0.56, -0.24]
Koltka 2011	2	0.5	30	4	1.25	30	20.0%	-2.00 [-2.48, -1.52]
Saritas 2015	0.5	0.7	30	0.6	0.5	30	21.7%	-0.10 [-0.41, 0.21]
**Subtotal (95% CI)**			**145**			**144**	**100.0%**	**-0.90 [-1.49, -0.30]**
Heterogeneity: Tau² = 0.40; Chi² = 59.78, df = 4 (P < 0.00001); I² = 93%								
Test for overall effect: Z = 2.95 (P = 0.003)								

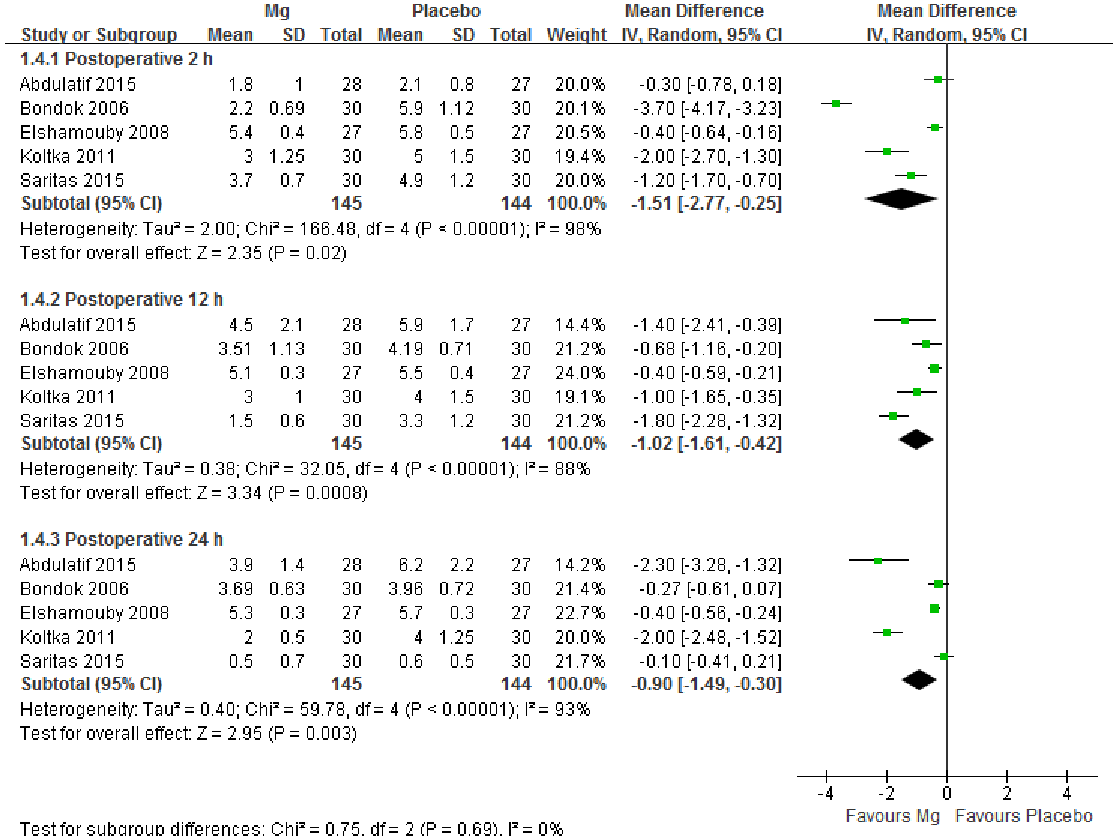

Test for subgroup differences: Chi² = 0.75, df = 2 (P = 0.69), I² = 0%

Figure 1 Forest plot of pain intensity at different follow-up time points between Mg and placebo.

Study or Subgroup	Mg Mean	Mg SD	Mg Total	Bupivacaine Mean	Bupivacaine SD	Bupivacaine Total	Weight	Mean Difference IV, Random, 95% CI
**2.4.1 Postoperative 1 h**								
Elshamouby 2008	5	0.6	27	3.9	0.4	27	35.0%	1.10 [0.83, 1.37]
Koltka 2011	5	1.5	30	3	1.75	30	30.7%	2.00 [1.18, 2.82]
Radwan 2013	2.6	0.7	20	3	0.6	20	34.3%	-0.40 [-0.80, 0.00]
**Subtotal (95% CI)**			**77**			**77**	**100.0%**	**0.86 [-0.36, 2.08]**
Heterogeneity: Tau² = 1.09; Chi² = 46.26, df = 2 (P < 0.00001); I² = 96%								
Test for overall effect: Z = 1.38 (P = 0.17)								
**2.4.2 Postoperative 2 h**								
Elshamouby 2008	5.4	0.4	27	4	0.3	27	35.5%	1.40 [1.21, 1.59]
Koltka 2011	3	1.25	30	2	2	30	29.9%	1.00 [0.16, 1.84]
Radwan 2013	2.3	0.6	20	2.4	0.5	20	34.7%	-0.10 [-0.44, 0.24]
**Subtotal (95% CI)**			**77**			**77**	**100.0%**	**0.76 [-0.37, 1.89]**
Heterogeneity: Tau² = 0.93; Chi² = 56.60, df = 2 (P < 0.00001); I² = 96%								
Test for overall effect: Z = 1.32 (P = 0.19)								
**2.4.3 Postoperative 4 h**								
Elshamouby 2008	5.3	0.5	27	4.3	0.3	27	35.3%	1.00 [0.78, 1.22]
Koltka 2011	3	1	30	2	1.25	30	31.2%	1.00 [0.43, 1.57]
Radwan 2013	2.1	0.6	20	2.4	0.7	20	33.5%	-0.30 [-0.70, 0.10]
**Subtotal (95% CI)**			**77**			**77**	**100.0%**	**0.56 [-0.31, 1.44]**
Heterogeneity: Tau² = 0.55; Chi² = 31.60, df = 2 (P < 0.00001); I² = 94%								
Test for overall effect: Z = 1.27 (P = 0.21)								
**2.4.4 Postoperative 12 h**								
Elshamouby 2008	5.1	0.3	27	4.3	0.4	27	36.8%	0.80 [0.61, 0.99]
Koltka 2011	3	1	30	2	1.75	30	30.8%	1.00 [0.28, 1.72]
Radwan 2013	2.5	0.7	20	3.2	1.2	20	32.4%	-0.70 [-1.31, -0.09]
**Subtotal (95% CI)**			**77**			**77**	**100.0%**	**0.38 [-0.58, 1.33]**
Heterogeneity: Tau² = 0.63; Chi² = 22.04, df = 2 (P < 0.0001); I² = 91%								
Test for overall effect: Z = 0.77 (P = 0.44)								
**2.4.5 Postoperative 24 h**								
Elshamouby 2008	5.3	0.3	27	4.3	0.4	27	38.0%	1.00 [0.81, 1.19]
Koltka 2011	2	0.5	30	2	2	30	32.4%	0.00 [-0.74, 0.74]
Radwan 2013	2.8	1.4	20	3.5	1.6	20	29.6%	-0.70 [-1.63, 0.23]
**Subtotal (95% CI)**			**77**			**77**	**100.0%**	**0.17 [-0.88, 1.23]**
Heterogeneity: Tau² = 0.75; Chi² = 18.07, df = 2 (P = 0.0001); I² = 89%								
Test for overall effect: Z = 0.32 (P = 0.75)								

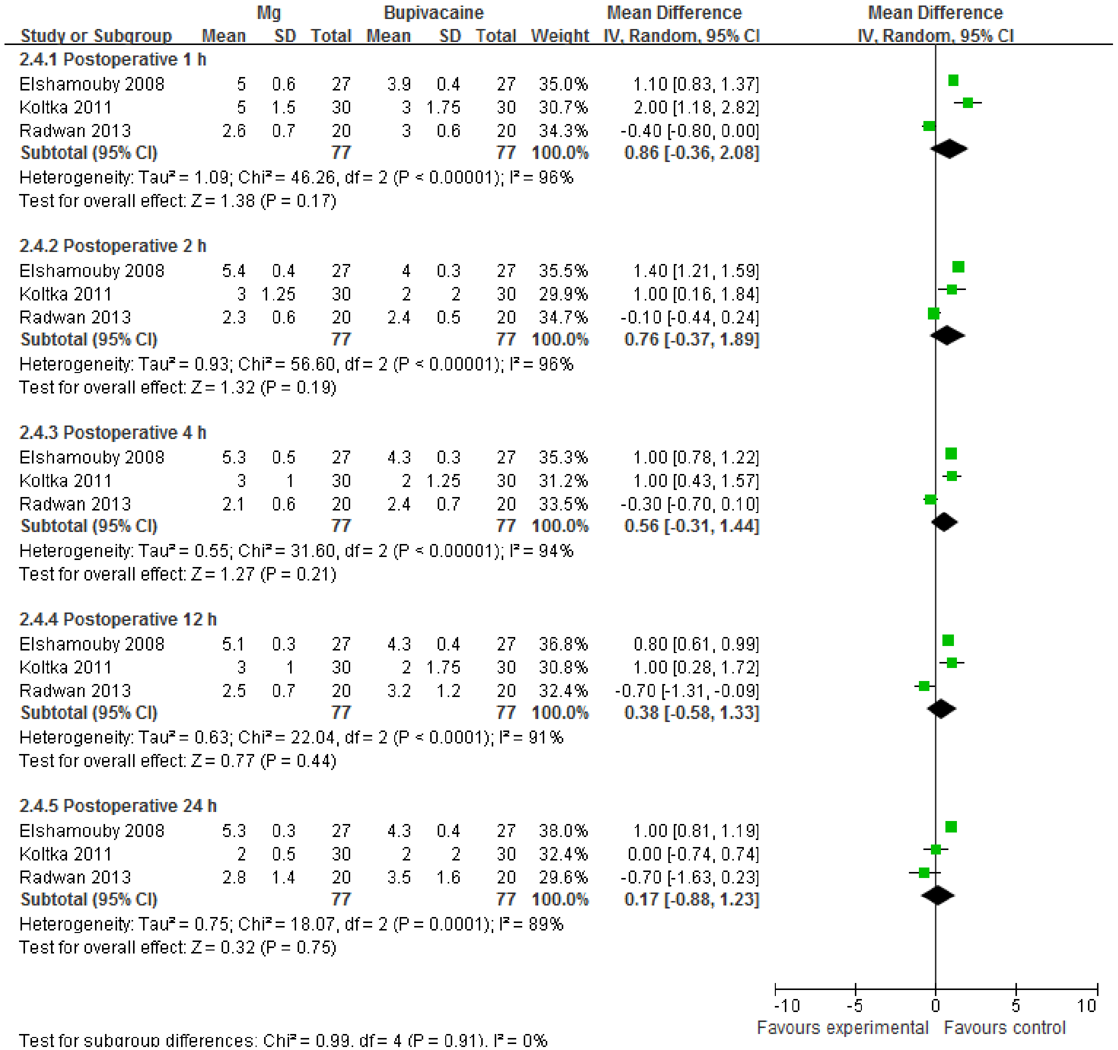

Test for subgroup differences: Chi² = 0.99, df = 4 (P = 0.91), I² = 0%

Figure 2 Forest plot of pain intensity at different follow-up time points between Mg and bupivacaine.

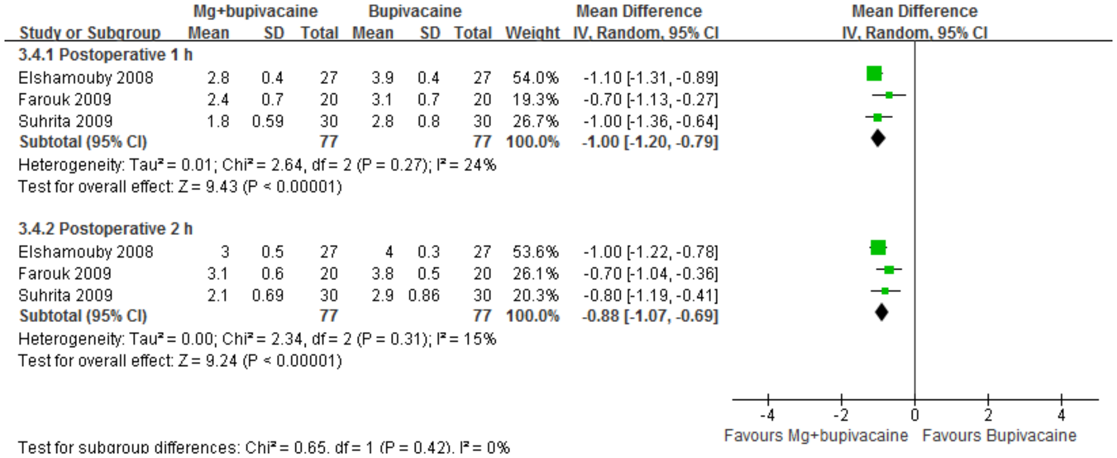

Study or Subgroup	Mg+bupivacaine Mean	Mg+bupivacaine SD	Mg+bupivacaine Total	Bupivacaine Mean	Bupivacaine SD	Bupivacaine Total	Weight	Mean Difference IV, Random, 95% CI
**3.4.1 Postoperative 1 h**								
Elshamouby 2008	2.8	0.4	27	3.9	0.4	27	54.0%	-1.10 [-1.31, -0.89]
Farouk 2009	2.4	0.7	20	3.1	0.7	20	19.3%	-0.70 [-1.13, -0.27]
Suhrita 2009	1.8	0.59	30	2.8	0.8	30	26.7%	-1.00 [-1.36, -0.64]
**Subtotal (95% CI)**			**77**			**77**	**100.0%**	**-1.00 [-1.20, -0.79]**
Heterogeneity: Tau² = 0.01; Chi² = 2.64, df = 2 (P = 0.27); I² = 24%								
Test for overall effect: Z = 9.43 (P < 0.00001)								
**3.4.2 Postoperative 2 h**								
Elshamouby 2008	3	0.5	27	4	0.3	27	53.6%	-1.00 [-1.22, -0.78]
Farouk 2009	3.1	0.6	20	3.8	0.5	20	26.1%	-0.70 [-1.04, -0.36]
Suhrita 2009	2.1	0.69	30	2.9	0.86	30	20.3%	-0.80 [-1.19, -0.41]
**Subtotal (95% CI)**			**77**			**77**	**100.0%**	**-0.88 [-1.07, -0.69]**
Heterogeneity: Tau² = 0.00; Chi² = 2.34, df = 2 (P = 0.31); I² = 15%								
Test for overall effect: Z = 9.24 (P < 0.00001)								

Test for subgroup differences: Chi² = 0.65, df = 1 (P = 0.42), I² = 0%

Figure 3 Forest plot of pain intensity at different follow-up time points between Mg plus bupivacaine and bupivacaine alone.
